# Impact of Concomitant Spine Comorbidities on Patient-Reported Outcomes After Total Knee Arthroplasty

**DOI:** 10.2106/JBJS.OA.26.00180

**Published:** 2026-07-13

**Authors:** Jisoo Lee, Heeyoon Chung, Won Seok Lee, Chong Bum Chang

**Affiliations:** 1 Department of Orthopaedic Surgery, Seoul National University Bundang Hospital, Seongnam, Republic of Korea; 2 Department of Orthopaedic Surgery, Geo-in Hospital, Busan, Republic of Korea; 3 Department of Orthopaedic Surgery, Inje University Sanggye Paik Hospital, Inje University College of Medicine, Seoul, Republic of Korea; 4 Department of Orthopaedic Surgery, Seoul National University College of Medicine, Seoul, Republic of Korea

## Abstract

**Background:**

The impact of clinically symptomatic spine comorbidities on patient-reported outcomes after total knee arthroplasty (TKA) remains unclear. We hypothesized that patients with pre-TKA spine comorbidities would demonstrate inferior outcomes compared with those without spine comorbidities.

**Methods:**

We retrospectively reviewed 492 patients who underwent primary TKA performed between 2020 and 2022. Spine comorbidities were defined as a history of lumbar injection, spine surgery, low back pain, or radiating pain. Three propensity score–matched analyses were performed: (1) primary analysis comparing patients with pre-TKA spine comorbidities vs. controls (141 pairs; 1:1 matching), (2) secondary analysis excluding patients who developed post-TKA spine comorbidities to isolate the effect of preexisting spine conditions (127 pairs; 1:1 matching), and (3) subgroup analysis comparing patients with prior spine surgery vs. those without (47 vs. 138; 1:3 matching). The Knee injury and Osteoarthritis Outcome Score (KOOS) was assessed preoperatively and at 1 and 2 years postoperatively.

**Results:**

There were 282 patients (141 pairs) in the primary analysis cohort (mean age, 72 years; 85% female). KOOS Sport showed the most consistent between-group differences across analyses. In the primary analysis, KOOS Sport was significantly lower in the spine comorbidity group at 2 years after false discovery rate (FDR) correction (mean, 43.4 vs. 53.9; mean difference, −10.5; Cohen's d = −0.45). KOOS Total showed nominally lower scores, although this did not remain significant after multiplicity adjustment. In the secondary analysis, KOOS Symptoms (71.2 vs. 78.0), Sport (44.1 vs. 56.5), and Total scores (66.4 vs. 72.9) were significantly lower at 2 years after FDR correction. In the subgroup analysis, patients with prior spine surgery showed lower KOOS scores in unadjusted comparisons at 2 years, although none of these differences remained significant after FDR correction.

**Conclusions:**

Pre-TKA spine comorbidities are associated with inferior KOOS outcomes, most consistently in high-demand activities. Awareness of these coexisting conditions may be helpful when counseling patients undergoing TKA about realistic expectations for postoperative functional recovery.

**Level of Evidence:**

Level III, Therapeutic Study. See Instructions for Authors for a complete description of levels of evidence.

## Introduction

Total knee arthroplasty (TKA) is one of the most successful surgical procedures for end-stage knee osteoarthritis, providing substantial pain relief and functional improvement for most patients^1-3^. However, patient satisfaction and postoperative function are influenced by factors beyond the knee joint itself^4-9^. Concurrent musculoskeletal conditions, particularly spine comorbidities, may affect recovery and limit the expected benefits of TKA^10-12^. Spinal comorbidities are highly prevalent among candidates for TKA because of shared risk factors such as advanced age, obesity, and generalized degenerative changes^13,14^. Although the concepts of “hip–spine syndrome” and “knee-spine syndrome” have highlighted the close interplay between lower limb joints and spine pathology^15-17^, the impact of coexisting spinal comorbidities on outcomes after TKA remains less clearly defined. Several mechanisms have been proposed by which spine comorbidities may affect post-TKA outcomes, including interaction between low back pain (LBP) and knee pain, the association between lumbar lordosis and knee extension limitation, and a link between spinal sagittal imbalance and knee range of motion^18-20^.

Previous studies have investigated the influence of radiographic lumbar spondylosis on TKA outcomes^13,21^. However, radiographic severity often correlates poorly with clinical symptoms and functional impairment^22,23^. Furthermore, there is limited evidence regarding the impact of clinically defined spinal comorbidities, characterized by active symptoms and treatment history rather than imaging findings alone, on patient-reported outcomes after TKA^12,24,25^. Therefore, the purpose of this study was to evaluate the association between pre-TKA spinal comorbidities and patient-reported outcomes. We hypothesized that patients with pre-TKA spinal comorbidities would demonstrate inferior patient-reported outcomes compared with those without spinal comorbidities, particularly in higher-demand functional domains requiring coordinated trunk and lower-limb movement.

## Materials and Methods

### Study Design and Patient Selection

This retrospective cohort study was approved by the Institutional Review Board of Seoul National University Bundang Hospital (IRB No. B-2602-1028-103). The requirement for informed consent was waived due to the retrospective nature of the study.

**Fig. 1:**
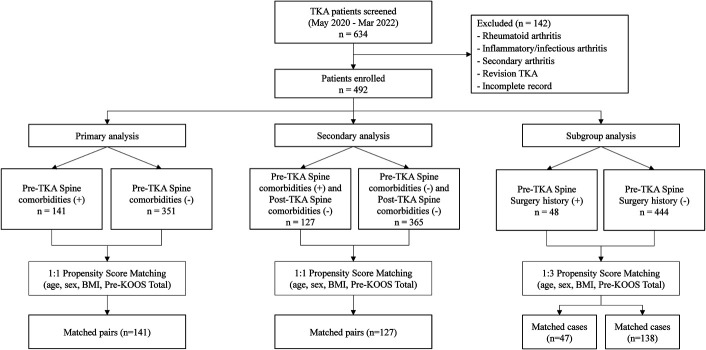
Flow diagram of patient selection and propensity score matched cohorts. Patients were categorized into primary, secondary, and subgroup analyses based on their spine comorbidity status. Propensity score matching was performed using age, sex, BMI, and preoperative KOOS Total score. BMI = body mass index, KOOS = Knee injury and Osteoarthritis Outcome Score, and TKA = total knee arthroplasty.

We screened 634 consecutive patients who underwent primary TKA for knee osteoarthritis at a single tertiary referral center between May 2020 and March 2022. Patients with rheumatoid, inflammatory, infectious, or secondary arthritis, revision TKA, or incomplete records were excluded. After exclusions, 492 patients were enrolled (Fig. 1). Three matched analyses were performed. Baseline characteristics of the primary matched cohort are summarized in Table I.

**TABLE I:** Baseline Characteristics of the Primary Matched Cohort: Patients with Pre-TKA Spine Comorbidities vs. Controls

Variable	Spine Comorbidity Group (n = 141)	Control Group (n = 141)	P	SMD
N (patients)	141	141	-	-
Age (yr)	72.33 ± 6.11	71.50 ± 5.58	0.239	0.141
Sex, female	120 (85.1%)	120 (85.1%)	1.000	-
BMI (kg/m^2^)	26.98 ± 3.54	26.69 ± 3.05	0.464	0.087
Pre-TKA KOOS				
Symptom	51.95 ± 18.67	53.50 ± 18.17	0.482	−0.084
Pain	43.70 ± 16.31	45.35 ± 15.11	0.378	−0.105
ADL	40.87 ± 17.81	44.24 ± 15.30	0.090	−0.203
Sport	10.99 ± 13.85	13.44 ± 14.33	0.146	−0.174
QoL	26.37 ± 13.51	27.88 ± 12.95	0.340	−0.114
Total	34.78 ± 12.78	36.88 ± 11.94	0.154	−0.170

ADL = activities of daily living, BMI = body mass index, KOOS = knee injury and osteoarthritis outcome score, QoL = quality of life, SMD = standardized mean difference, and TKA = total knee arthroplasty.

Values are presented as mean ± SD or number (%). P-values were calculated using the independent *t* test for continuous variables and the χ^2^ test for categorical variables. Propensity score matching was performed in a 1:1 ratio based on age, sex, BMI, and preoperative KOOS total score.

All surgeries were performed by a single surgeon with a standardized technique and rehabilitation pathway. The study period was chosen to ensure a minimum 2-year follow-up while preserving consecutive enrollment.

### Definition of Pre-TKA Spine Comorbidities

Pre-TKA spine comorbidities were defined as the presence of at least one of the following 4 factors identified through a comprehensive review of electronic medical records before TKA: (1) history of lumbar spinal injection, (2) history of lumbar spine surgery, (3) LBP, and (4) radiating pain (RP) in lower extremities. The distribution of these components in the matched primary-analysis cohort is summarized in Appendix Table 1. Patients with none of these factors were classified as controls. Among patients with pre-TKA spine comorbidities, those with a history of spine surgery were further analyzed as a subgroup. Types of spine surgery included spinal fusion, decompression, discectomy, and vertebroplasty (Appendix Table 2).

### Propensity Score Matching

To reduce selection bias, propensity score matching (PSM) was performed. Propensity scores were calculated using logistic regression with age, sex, body mass index (BMI), and preoperative Knee injury and Osteoarthritis Outcome Score (KOOS) Total as covariates. Nearest neighbor matching with a caliper width of 0.2 standard deviations of the logit of the propensity score was used, as recommended by Austin^26^. Balance between groups was assessed using standardized mean differences (SMD), with SMD <0.2 considered acceptable balance.

### Outcome Assessment

The KOOS^27,28^ was used to evaluate patient-reported outcomes. The KOOS consists of 5 subscales: Symptoms, Pain, Activities of Daily Living (ADL), Sport and Recreation (Sport), and Quality of Life (QoL). Each subscale is scored from 0 to 100, with higher scores indicating better outcomes. The KOOS Total score was calculated as the mean of the 5 subscales. KOOS was assessed preoperatively and at 1 and 2 years after TKA.

### Statistical Analysis

We conducted 3 sets of analyses. In the primary analysis (Table II), patients were grouped based solely on pre-TKA spine comorbidity status and post-TKA spine comorbidity status was not considered. In the secondary analysis (Table III), only patients without post-TKA spine comorbidities were included in both groups to isolate the effect of pre-existing spine conditions. In the subgroup analysis (Table IV), patients with pre-TKA spine surgery were compared with those without pre-TKA spine surgery history. In both groups, those who underwent spine surgery after TKA were excluded. Patients were matched 1:1 in the primary and secondary analyses and 1:3 in the subgroup analysis to maintain adequate statistical power.

**TABLE II:** Comparison of Patient-Reported Outcomes (KOOS) in the Primary Analysis: Impact of Pre-TKA Spine Comorbidities

KOOS Subscale		Spine Comorbidity Group (n = 141)	Control Group (n = 141)	Mean Difference (95% CI)	Cohen's d (95% CI)	P	P (FDR)
Symptom	Pre-TKA	51.95 ± 18.67	53.50 ± 18.17	−1.55 (−5.86, 2.77)	−0.08 (−0.32, 0.15)	0.482	-
	1 yr post-TKA	73.91 ± 14.45	76.69 ± 15.09	−2.78 (−6.49, 0.93)	−0.19 (−0.44, 0.06)	0.142	0.253
	2 yr post-TKA	72.50 ± 17.43	76.18 ± 16.82	−3.68 (−8.68, 1.31)	−0.22 (−0.51, 0.08)	0.148	0.253
Pain	Pre-TKA	43.70 ± 16.31	45.35 ± 15.11	−1.65 (−5.34, 2.03)	−0.11 (−0.34, 0.13)	0.378	-
	1 yr post-TKA	79.16 ± 16.55	81.11 ± 14.77	−1.96 (−5.90, 1.99)	−0.12 (−0.37, 0.13)	0.330	0.390
	2 yr post-TKA	81.26 ± 15.13	83.28 ± 14.45	−2.02 (−6.33, 2.30)	−0.14 (−0.43, 0.15)	0.357	0.390
ADL	Pre-TKA	40.87 ± 17.81	44.24 ± 15.30	−3.37 (−7.26, 0.52)	−0.20 (−0.44, 0.03)	0.090	-
	1 yr post-TKA	76.31 ± 16.28	79.41 ± 14.47	−3.10 (−6.97, 0.77)	−0.20 (−0.45, 0.05)	0.116	0.253
	2 yr post-TKA	78.90 ± 13.41	80.99 ± 16.17	−2.08 (−6.39, 2.22)	−0.14 (−0.43, 0.15)	0.341	0.390
Sport	Pre-TKA	10.99 ± 13.85	13.44 ± 14.33	−2.45 (−5.75, 0.86)	−0.17 (−0.41, 0.06)	0.146	-
	1 yr post-TKA	37.23 ± 23.73	45.88 ± 25.34	−8.65 (−14.81 to −2.49)	−0.35 (−0.60 to −0.10)	**0.006**	**0.037**
	2 yr post-TKA	43.39 ± 23.59	53.92 ± 23.65	−10.53 (−17.41 to −3.64)	−0.45 (−0.74 to −0.15)	**0.003**	**0.035**
QoL	Pre-TKA	26.37 ± 13.51	27.88 ± 12.95	−1.51 (−4.61, 1.60)	−0.11 (−0.35, 0.12)	0.340	-
	1 yr post-TKA	54.65 ± 19.76	56.65 ± 19.66	−2.00 (−6.95, 2.95)	−0.10 (−0.35, 0.15)	0.427	0.427
	2 yr post-TKA	56.68 ± 20.35	60.50 ± 19.19	−3.82 (−9.59, 1.95)	−0.19 (−0.48, 0.10)	0.193	0.290
Total	Pre-TKA	34.78 ± 12.78	36.88 ± 11.94	−2.10 (−5.00, 0.79)	−0.17 (−0.40, 0.06)	0.154	-
	1 yr post-TKA	64.25 ± 14.72	67.95 ± 14.10	−3.70 (−7.32 to −0.07)	−0.26 (−0.51 to −0.01)	**0.045**	0.136
	2 yr post-TKA	66.55 ± 14.47	70.97 ± 14.62	−4.43 (−8.66 to −0.19)	−0.30 (−0.59 to −0.01)	**0.041**	0.136

ADL = activities of daily living, CI = confidence interval, FDR = false discovery rate, KOOS = knee injury and osteoarthritis outcome score, QoL = quality of life, and TKA, total knee arthroplasty.

Values are presented as mean ± SD. Mean difference was calculated by subtracting control group values from spine comorbidity group values. Cohen's d was calculated using pooled SD. Comparison between groups was performed using the independent *t* test. FDR-adjusted P-values were calculated using the Benjamini-Hochberg method. Propensity score matching was performed in a 1:1 ratio based on age, sex, BMI, and preoperative KOOS Total score. Bold values indicate statistical significance (P < 0.05).

**TABLE III:** Comparison of Patient-Reported Outcomes (KOOS) in the Secondary Analysis: Excluding Patients with Post-TKA Spine Comorbidities

KOOS Subscale		Spine Comorbidity Group (n = 127)	Control Group (n = 127)	Mean Difference (95% CI)	Cohen's d (95% CI)	P	P (FDR)
Symptom	Pre-TKA	51.60 ± 19.33	52.84 ± 18.67	−1.24 (−5.93, 3.46)	−0.07 (−0.31, 0.18)	0.604	-
	1 yr post-TKA	73.82 ± 13.86	76.77 ± 15.00	−2.95 (−6.76, 0.86)	−0.20 (−0.47, 0.06)	0.129	0.154
	2 yr post-TKA	71.20 ± 19.35	77.97 ± 16.35	−6.77 (−12.34 to −1.19)	−0.38 (−0.69 to −0.07)	**0.018**	**0.035**
Pain	Pre-TKA	44.58 ± 17.41	45.34 ± 15.25	−0.77 (−4.81, 3.28)	−0.05 (−0.29, 0.20)	0.710	-
	1 yr post-TKA	78.62 ± 15.61	82.51 ± 13.69	−3.89 (−7.76 to −0.01)	−0.26 (−0.53 to −0.00)	**0.049**	0.074
	2 yr post-TKA	81.05 ± 16.24	85.51 ± 12.87	−4.46 (−9.02, 0.10)	−0.31 (−0.61, 0.01)	0.055	0.074
ADL	Pre-TKA	40.92 ± 18.82	43.94 ± 15.99	−3.02 (−7.34, 1.29)	−0.17 (−0.42, 0.07)	0.169	-
	1 yr post-TKA	76.50 ± 15.37	81.13 ± 13.00	−4.64 (−8.39 to −0.88)	−0.33 (−0.59 to −0.06)	**0.016**	**0.035**
	2 yr post-TKA	77.83 ± 14.48	82.69 ± 15.25	−4.86 (−9.47 to −0.25)	−0.33 (−0.64 to −0.02)	**0.039**	0.067
Sport	Pre-TKA	12.87 ± 15.55	14.17 ± 14.98	−1.30 (−5.07, 2.47)	−0.09 (−0.33, 0.16)	0.498	-
	1 yr post-TKA	39.38 ± 23.91	48.29 ± 24.91	−8.91 (−15.36 to −2.47)	−0.37 (−0.63 to −0.10)	**0.007**	**0.028**
	2 yr post-TKA	44.05 ± 25.43	56.45 ± 24.26	−12.40 (−20.12 to −4.67)	−0.50 (−0.81 to −0.19)	**0.002**	**0.022**
QoL	Pre-TKA	25.94 ± 14.18	27.02 ± 13.17	−1.08 (−4.46, 2.30)	−0.08 (−0.33, 0.17)	0.529	-
	1 yr post-TKA	54.69 ± 20.64	57.88 ± 19.08	−3.20 (−8.44, 2.05)	−0.16 (−0.42, 0.10)	0.231	0.231
	2 yr post-TKA	57.59 ± 21.71	61.82 ± 18.60	−4.23 (−10.52, 2.06)	−0.21 (−0.52, 0.10)	0.186	0.203
Total	Pre-TKA	35.18 ± 14.07	36.66 ± 12.49	−1.48 (−4.77, 1.81)	−0.11 (−0.36, 0.13)	0.376	-
	1 yr post-TKA	64.60 ± 14.29	69.32 ± 13.13	−4.72 (−8.34 to −1.10)	−0.34 (−0.61 to −0.08)	**0.011**	**0.033**
	2 yr post-TKA	66.35 ± 16.43	72.89 ± 13.80	−6.54 (−11.26 to −1.82)	−0.43 (−0.74 to −0.12)	**0.007**	**0.028**

ADL = activities of daily living, CI = confidence interval, FDR = false discovery rate, KOOS = knee injury and osteoarthritis outcome score, QoL = quality of life, TKA = total knee arthroplasty.

Values are presented as mean ± SD. Mean difference was calculated by subtracting control group values from spine comorbidity group values. Cohen's d was calculated using pooled SD. Comparison between groups was performed using the independent *t* test. FDR-adjusted P-values were calculated using the Benjamini-Hochberg method. Propensity score matching was performed in a 1:1 ratio based on age, sex, BMI, and preoperative KOOS Total score. Patients who developed spine comorbidities after TKA were excluded. Baseline characteristics were well balanced (Appendix Table 1). Bold values indicate statistical significance (P < 0.05).

**TABLE IV:** Comparison of Patient-Reported Outcomes (KOOS) in the Subgroup Analysis: Patients with Prior Spine Surgery vs. Controls

KOOS Subscale		Prior Spine Surgery Group (n = 47)	Control Group (n = 138)	Mean Difference (95% CI)	Cohen's d (95% CI)	P	P (FDR)
Symptom	Pre-TKA	46.81 ± 19.66	48.16 ± 19.74	−1.35 (−7.97, 5.26)	−0.07 (−0.40, 0.26)	0.685	-
	1 yr post-TKA	65.75 ± 13.95	71.07 ± 15.30	−5.31 (−10.58, −0.05)	−0.35 (−0.72, 0.01)	**0.048**	0.082
	2 yr post-TKA	69.43 ± 15.90	71.54 ± 17.43	−2.11 (−8.64, 4.42)	−0.12 (−0.52, 0.27)	0.521	0.521
Pain	Pre-TKA	38.89 ± 16.98	41.79 ± 17.07	−2.90 (−8.61, 2.82)	−0.17 (−0.50, 0.16)	0.316	-
	1 yr post-TKA	69.59 ± 20.47	79.05 ± 15.27	−9.46 (−16.62 to −2.31)	−0.57 (−0.93 to −0.20)	**0.011**	0.082
	2 yr post-TKA	78.35 ± 19.11	83.27 ± 13.82	−4.92 (−12.12, 2.27)	−0.32 (−0.71, 0.07)	0.175	0.191
ADL	Pre-TKA	35.70 ± 15.21	36.89 ± 17.43	−1.19 (−6.49, 4.11)	−0.07 (−0.40, 0.26)	0.656	-
	1 yr post-TKA	69.34 ± 19.75	75.19 ± 15.51	−5.84 (−12.80, 1.11)	−0.35 (−0.71, 0.01)	0.098	0.147
	2 yr post-TKA	72.06 ± 19.25	80.13 ± 15.33	−8.07 (−15.43 to −0.72)	−0.49 (−0.89 to −0.09)	**0.032**	0.082
Sport	Pre-TKA	14.47 ± 17.48	9.24 ± 11.85	5.23 (−0.26, 10.71)	0.39 (0.05, 0.72)	0.061	-
	1 yr post-TKA	33.72 ± 22.85	40.55 ± 23.59	−6.83 (−15.33, 1.66)	−0.29 (−0.65, 0.07)	0.113	0.151
	2 yr post-TKA	41.03 ± 25.73	51.65 ± 24.54	−10.62 (−20.81 to −0.43)	−0.43 (−0.82 to −0.03)	**0.041**	0.082
QoL	Pre-TKA	26.20 ± 16.10	23.28 ± 12.97	2.92 (−2.26, 8.10)	0.21 (−0.12, 0.54)	0.265	-
	1 yr post-TKA	50.00 ± 19.08	55.08 ± 19.13	−5.08 (−12.13, 1.96)	−0.27 (−0.63, 0.10)	0.154	0.185
	2 yr post-TKA	54.04 ± 17.54	62.57 ± 19.99	−8.52 (−15.80 to −1.24)	−0.44 (−0.84 to −0.04)	**0.022**	0.082
Total	Pre-TKA	32.41 ± 14.54	31.87 ± 12.21	0.54 (−4.17, 5.25)	0.04 (−0.29, 0.37)	0.820	-
	1 yr post-TKA	57.68 ± 15.97	64.19 ± 13.86	−6.51 (−12.23 to −0.79)	−0.45 (−0.82 to −0.09)	**0.027**	0.082
	2 yr post-TKA	62.98 ± 16.77	69.83 ± 14.62	−6.85 (−13.37 to −0.33)	−0.45 (−0.85 to −0.05)	**0.040**	0.082

ADL, Activities of Daily Living; CI, confidence interval; FDR, false discovery rate; KOOS, Knee injury and Osteoarthritis Outcome Score; QoL, Quality of Life; SD, standard deviation; TKA, total knee arthroplasty

Values are presented as mean ± SD. Mean difference was calculated by subtracting control group values from prior spine surgery group values. Cohen's d was calculated using pooled SD. Comparison between groups was performed using the independent t-test. FDR-adjusted P-values were calculated using the Benjamini-Hochberg method. Propensity score matching was performed in a 1:3 ratio based on age, sex, BMI, and preoperative KOOS Total score. Patients who underwent spine surgery after TKA were excluded. Baseline characteristics were well balanced (Appendix Table 2). Bold values indicate statistical significance (P < 0.05).

Statistical analysis was performed using R version 4.5.1 (R Foundation for Statistical Computing, Vienna, Austria). Continuous variables were expressed as mean ± SD and compared with independent *t* tests at each time point. As a supplementary analysis, linear mixed-effects models (LMM) included group, time, and group × time as fixed effects and patient as a random intercept (Appendix Table 3). Categorical variables were compared with chi-square or Fisher exact tests. A p-value of <0.05 was considered statistically significant. Benjamini-Hochberg false discovery rate (FDR) correction was applied to postoperative comparisons; both unadjusted and adjusted p-values were reported. No a priori sample size calculation was performed because this was a retrospective cohort study with consecutive enrollment.

## Results

### Patient Characteristics

Of the 634 patients screened, 492 were enrolled; 141 (28.7%) had pre-TKA spine comorbidities and 351 (71.3%) did not.

In the primary analysis, 141 pairs were matched, with no significant baseline differences in age, sex, BMI, or preoperative KOOS scores (Table I). KOOS Sport subscale was significantly lower in the spine comorbidity group at both 1 and 2 years after TKA, and this finding remained significant after FDR correction (Table II). KOOS Total showed nominally lower scores at 1 and 2 years (unadjusted p = 0.045 and 0.041, respectively), although these did not remain significant after multiplicity adjustment. No significant differences were observed in Symptom, Pain, ADL, or QoL subscales.

In the secondary analysis, 127 pairs were included, with well-balanced baseline characteristics (Appendix Table 4). At 2 years after TKA, KOOS Sport, Symptom, and Total were lower in the spine comorbidity group after FDR correction (Table III). KOOS ADL was significantly lower at 1 year (FDR-adjusted p = 0.035) but did not remain significant at 2 years after correction (FDR-adjusted p = 0.067). Pain subscale approached significance (p = 0.055).

In the subgroup analysis, 47 patients and 138 controls were included after 1:3 matching, with well-balanced baseline characteristics (Appendix Table 5). Unadjusted 2-year differences were observed in KOOS ADL, Sport, QoL, and Total, but none remained significant after FDR correction. (Table IV).

Supplementary LMM analyses showed significant group × time interactions for KOOS Sport across all 3 analyses (Appendix Table 3).

## Discussion

The principal findings of this study were that patients with pre-TKA spine comorbidities demonstrated inferior KOOS outcomes at 2 years following TKA compared with matched controls. After 1:1 PSM for age, sex, BMI, and preoperative KOOS Total, the spine-comorbidity group showed the most consistent differences in the KOOS Sport subscale, supporting our hypothesis that higher-demand functional domains would be most affected. These differences were more pronounced when patients who developed post-TKA spine comorbidities were excluded, with additional differences observed in Symptom, ADL, and Total scores. In the exploratory subgroup analysis of patients with prior spine surgery, unadjusted differences were observed but did not remain significant after multiplicity adjustment.

Supplementary LMM analyses showed significant group × time interactions for KOOS Sport, suggesting that the between-group divergence widened over time.

The differential impact across KOOS subscales provides important mechanistic insights. The Sport subscale, which assesses high-demand activities such as squatting, kneeling, jumping, running, and pivoting, showed the largest between-group differences. In the secondary analysis, the 2-year between-group difference was 12.4 points with a moderate effect size (Cohen’s d = −0.50; 95% confidence interval, −0.81 to −0.19), suggesting potentially clinically relevant limitation in higher-demand function. A plausible explanation, consistent with prior studies on lumbopelvic-knee biomechanical interactions, is that these activities require coordinated lower limb and trunk movement and may therefore be more susceptible to the combined effects of knee and spine disease^19,20,29-32^, although this was not directly assessed in the current study. In contrast, Pain, ADL, and QoL subscales showed smaller differences, suggesting that TKA provides substantial pain relief and basic functional recovery regardless of spine comorbidity status, although prexisting spine pathology may still exert a measurable effect on these domains. Smaller differences in these subscales should be interpreted more cautiously.

Our findings align with previous studies that have identified an association between spine pathology and inferior outcomes after lower extremity arthroplasty. Our earlier research demonstrated that radiographic lumbar spondylosis was highly prevalent among TKA patients, and that severe RP during activity exerts adverse effects on clinical outcomes after TKA^13^. This study extends this understanding by employing a comprehensive definition of spine comorbidities that includes both treatment history and symptom-based criteria, thereby capturing a broader spectrum of clinically significant spine pathology. Unlike radiographic findings alone, which often correlate poorly with functional impairment^23,33^, this multifactorial definition successfully identified patients at risk for suboptimal outcomes, particularly in high-demand activities.

Several studies have explored the relationships between the spine and the major lower-limb joints, including the hip and knee. Studies on the hip–spine relationship have shown that abnormal spinopelvic alignment or unrecognized lumbar disease can adversely affect the outcomes of total hip arthroplasty, including instability and persistent pain^34,35^. In the knee, the concept of a “knee–spine syndrome” has been used to describe the association between lumbar alignment and knee flexion deformity or patellofemoral pain^19^. It was reported that concomitant LBP is an independent predictor of worse patient-reported outcomes and lower satisfaction after TKA^10-12^, which is consistent with our finding that spinal comorbidities are associated with inferior KOOS scores, particularly in sport and recreational activities.

Although the subgroup analysis of patients with prior spine surgery did not yield statistically significant differences after multiplicity adjustment, this group likely represents a population with more severe or refractory pathology, potentially with residual structural or neurological deficits. This may explain the sustained functional limitations observed 2 years after TKA. Among patients with prior spine surgery, spinal fusion (n = 28) and decompression (n = 17) were the most common procedures (Appendix Table 2). In particular, patients with prior spinal fusion showed lower Sport scores at 2 years post-TKA compared with those who underwent decompression alone (35.45 vs. 57.22, p = 0.046, Appendix Table 6). The comparison between fusion and decompression was based on small samples, limiting statistical power and precision. Therefore, these findings should be interpreted as exploratory. This difference may be attributed to 2 factors: first, spinal fusion is typically indicated for more severe pathology involving mechanical instability, suggesting a worse baseline spine condition; second, fusion surgery itself results in loss of segmental motion and increased spinal rigidity, which may further limit the patient's ability to perform high-demand physical activities that require trunk flexibility and coordination.

These findings encourage preoperative counseling regarding concomitant spine comorbidities. Patients with pre-TKA spine comorbidities may benefit from understanding that while TKA will provide substantial pain relief and improvement in daily activities, their capacity for high-demand activities may remain limited.

This study has several limitations. First, as a retrospective single-center study, selection bias cannot be excluded despite consecutive enrollment and PSM. Potential confounders such as psychological factors and lower-extremity alignment were not measured and may contribute residual confounding. The single-surgeon, standardized rehabilitation design reduced but did not eliminate perioperative variability. Second, our definition of pre-TKA spine comorbidities was intentionally inclusive to capture the heterogeneous presentation of spine-origin problems, but this may have introduced clinical heterogeneity within the exposure group; conversely, a narrower definition would risk excluding symptomatic patients. Third, the subgroup analyses were based on relatively small sample sizes, limiting statistical power and precision for smaller between-group differences; these findings should therefore be interpreted cautiously. Fourth, reliance on medical record documentation may have not captured subclinical or unreported spine pathology, and spine-specific instruments such as the Oswestry Disability Index were not used^36^. Fifth, although we characterized the clinical components of spine comorbidity and fusion levels, more detailed diagnostic subclassification could not be systematically determined from the available records. Finally, the 2-year follow-up period, while adequate for assessing medium-term outcomes, may not capture longer-term recovery trajectories or late manifestation of spine-related limitations.

In conclusion, patients with pre-TKA spine comorbidities demonstrate inferior patient-reported outcomes at 2 years after TKA compared with those without spine comorbidities. Although TKA yields substantial improvement in pain and daily function regardless of spine comorbidity status, recovery of higher-demand activities appears more limited in the presence of concurrent spine pathology. These findings suggest that recognition of concomitant spine comorbidities may help clinicians manage patient expectations regarding postoperative functional recovery, particularly for higher-demand activities.

## Funding

No external funding was received for this study.

## Appendix

Supporting material provided by the authors is posted with the online version of this article as a data supplement at jbjs.org (http://links.lww.com/JBJSOA/B275). This content was not copy edited or verified by JBJS.
